# Multisensory stimulation and its effect on breast milk volume production in mothers of premature infants

**DOI:** 10.3389/fped.2024.1331310

**Published:** 2024-03-14

**Authors:** Carla Madeleine Cuya, Carlos Barriga, Maria del Carmen Graf, Mirta Cardeña, María del Pilar Borja, Richard Condori, Moises Azocar, Carlos Cuya

**Affiliations:** ^1^ Nursing Faculty, Universidad Católica de Santa María, Arequipa, Peru; ^2^ Social Sciences and Technologies and Humanities Faculty, Universidad Católica de Santa María, Arequipa, Peru; ^3^ Nursing, University of Wisconsin Oshkosh, Oshkosh, WI, United States; ^4^ Medicine Faculty, Universidad Católica de Santa María, Arequipa, Peru; ^5^ Manuel de Torres Muñoz Hospital (EsSalud), Arequipa, Peru

**Keywords:** breastfeeding, premature birth, emotions, sensory system, limbic systems

## Abstract

**Introduction:**

In a significant number of NICUs, mothers are unable to provide enough maternal milk to feed their premature babies, so healthcare workers rely on human milk banks. Unfortunately, this service is not available in many countries, such as Peru, where premature infants receive formula. The aim of this study was to determine the effectiveness of multisensory stimulation on mother's own milk production.

**Methods:**

Participants in this study were postpartum mothers of preterm infants 27–37 weeks gestational age. The participants were assigned to three groups: (1) audiovisual stimulation (SAV) (*n* = 17), (2) audiovisual and olfactory stimulation (SAVO) (*n* = 17), and (3) control (*n* = 16). A questionnaire was used to collect demographic and obstetric data, including a record of mother's own milk volume.

**Results:**

There was no significant difference between the SAV, SAVO and control groups regarding age, marital status, education level, occupation, number of children, mode of delivery, Apgar and birth weight. On the other hand, a significant difference was observed between the SAV and SAVO groups regarding the amount of milk produced, with higher production between the fourth and seventh day (Tukey *p* < 0.05). Similarly, milk volume was significantly greater in the SAVO group compared to the SAV and control groups (OR = 1.032, 95% CI = 1.0036–1.062, *p* < 0.027).

**Conclusion:**

Multisensory stimulation in postpartum mothers of preterm infants caused an increase in the volume of mother's own milk production. However, more research is needed to explain the findings presented in this study.

## 1 Introduction

Mother's own milk is undoubtedly the ideal and first food to promote the healthy growth and development of newborns, constituting a fundamental pillar at the beginning of their lives (1). It is rich in essential nutrients such as proteins, fats, carbohydrates, vitamins and minerals that promote the mental and physical development of premature infants (2). Therefore, the implementation of policies that actively encourage and promote mother's own milk feeding in this context becomes an unavoidable priority for health institutions (3, 4). Although preterm infants face developmental challenges due to their physiological immaturity, making them susceptible to various health conditions (5), mother's own milk acts as a protective factor due to its abundant immunoglobulins and other bioactive components (6).

One of the most common conditions faced by premature infants is necrotizing enterocolitis (2), an inflammation of the colon that occurs in response to the premature development of the digestive system. Necrotizing enterocolitis can have serious and, in some cases, life-threatening consequences, underscoring the importance of ensuring adequate nutrition from the first moments of life. Despite this, several neonatal intensive care units (NICUs) face a critical challenge: access and availability of mother's own milk (7).

Furthermore, the lack of availability of mother's own milk, in neonatal intensive care units, due to the fact that mothers often do not produce enough maternal milk (8), could be considered as a public health problem. Therefore, the unavailability of mother's own milk for immediate feeding of preterm infants, a common situation in many neonatal intensive care units (NICUs), leads health care providers to rely on donor human milk (2, 8, 9). In this case, the limited number of human milk banks aggravates the problem of neonatal care, as these centers could provide an alternative for feeding premature infants (10). Due to the difficulties in the implementation and operation of this type of basic health service, which could be attributed to the lack of an adequate government management and legal regulation in countries such as Peru (11–13). However, in the absence of a human milk bank, health care institutions feel the need to administer infant formula, which several studies have shown to be detrimental to infant growth and development (14–16). In the study by Moreira et al. (2) it was stated that the main complications of the use of formula in premature neonates in the NICU was the presence of necrotizing enterocolitis, which can be explained from the intestinal microbiota, where babies fed with mother's ow milk have a lower amount of enterobacteria than babies who receive formula or donor human milk. It also showed that babies fed with formula presented other complications, such as sepsis and bronchopulmonary dysplasia, unlike babies who received donor human milk or mother's own milk. This is because mother's own milk has metabolic advantages over formula, resulting in better protein absorption and more significant tissue growth in the newborn (17).

Another difference that has been observed between mother's own milk vs. formula, is the fat mass index, which is lower in newborns who are mother's own milk-fed than in those who are fed formula (17). Thus, mother's own milk should be the feeding source of choice for preterm infants.

In this context, the insufficient amount of milk produced by the mothers of preterm infants is one of the main reasons for the lack of mother's own milk in the NICU (8), a problem that may be due to multiple causes (12, 18). This condition can be explained by failed or delayed lactogenesis II, the second stage of milk production, which occurs after birth (18, 19). Underlying neurophysiological mechanisms such as endocrine, metabolic and obstetric factors are involved in lactogenesis II.

On the endocrine aspect, prolactin, insulin, adrenal cortisol and thyroid hormones can act directly on lactotropic cells and indirectly alter the endocrine response and the supply of nutrients to the mammary gland for milk production (20–22). In addition to these factors, oxytocin plays a pivotal role in both the synthesis and ejection of maternal milk. Oxytocin is synthesized in magnocellular neurons and primarily accumulates in the paraventricular nuclei (PVN) and supraoptic nuclei (23). Its presence extends from the median eminence internal zone to the neurohypophysis (24), as well as collateral projections to the central amygdala and nucleus accumbens (25).

In terms of metabolic and obstetric factors that may delay lactogenesis II in mothers of premature infants, various conditions should be considered, such as retained placenta, cesarean section, type 2 diabetes, gestational diabetes, childbirth stress, polycystic ovarian syndrome, and postpartum hemorrhage, among others (18). Obese women are also more likely to have delayed lactogenesis II due to hormonal influences on milk production (26). Limitations have also been described in the maintenance of breastfeeding in preterm infants (27), which may be due to the immaturity of the sucking and swallowing reflexes of the newborn, who cannot be fed directly from the mother's breast, requiring manual extraction and collection of milk for later delivery to health care personnel (20).

Regarding the problem of insufficient mother's own milk production, several studies have demonstrated the importance of intervening with external and environmental stimuli. For example, the study by Cohen et al. (28), in lactating female mice showed that exposure to the odor of their pups resulted in significant changes in the cortical area of the mice's brains, leading to improved mother-pup behavior. Similarly, Embarek-Hernández et al. (29), report that multisensory stimulation improves feeding behavior in children and may include visual, tactile, oral, vestibular, auditory, or kinesthetic stimulation. Other similar studies, such as Hernández-Gutiérrez et al. (30), show that a combination of tactile, kinesthetic, and oral stimulation stimulates feeding responses in preterm infants compared with oral stimulation alone.

The production of mother's own milk can be influenced by multisensory stimuli at the level of oxytocin neurons (16) in the central amygdala and nucleus accumbens (25). Therefore, multisensory stimuli could induce an emotional and sentimental response (amygdala and hypothalamus) (31), which would result in the production of oxytocin through the action of oxytocinergic nerves originating in the PVN (32), which increase the blood flow of milk to the nipple during breastfeeding (23).

To summarize, the present study aimed to determine the efficacy of these stimulating processes on milk production in mothers of preterm infants.

## 2 Materials and methods

### 2.1 Study design

The design of the present study corresponds to case–control study, in accordance with the CONSORT 2010 guidelines (33). The study was conducted in the facilities of the Hospital Goyeneche, located in the city of Arequipa, in the southern region of Peru. Study participants were postpartum mothers with preterm infants born between 27 and 37 weeks of gestation. The study period was between June and December 2022. It should be noted that during this period there were sanitary restrictions in all Peruvian health facilities due to the COVID-19 pandemic (34). Therefore, maternal contact with the newborns was minimal. Even the kangaroo mother care method could not be applied during the study period due to the health restrictions that existed at the time.

The Institutional Research Ethics Committee of the Catholic University of Santa Maria approved the research project in the city of Arequipa, Peru (code: 099-2022). Prior to data collection, the researchers provided all participants with a detailed verbal and written explanation of the objectives and scope of the study, the data collection process, and their rights and responsibilities. Written informed consent was obtained from all participants. All information obtained during the study was kept strictly confidential. The results were presented anonymously to prevent the disclosure of any personal information about the participants. Participants were also free to withdraw or terminate their participation in the study at any time. However, there were no dropouts during the study.

In the case of the inclusion criteria, the selection of participants was postpartum mothers with premature infants born within 27–37 weeks of gestation, who had not received galactagogues, who did not have comorbidity or health complications in the breasts, and who wished to participate in the study, as well as premature infants who did not have malformations or health complications, who were born with an Apgar score greater than 7, at 5 min, with a weight between 1,000 and 3,900 g. Candidates who did not meet the above criteria were excluded from the study.

### 2.2 Sampling and recruitment

**Figure 1:**
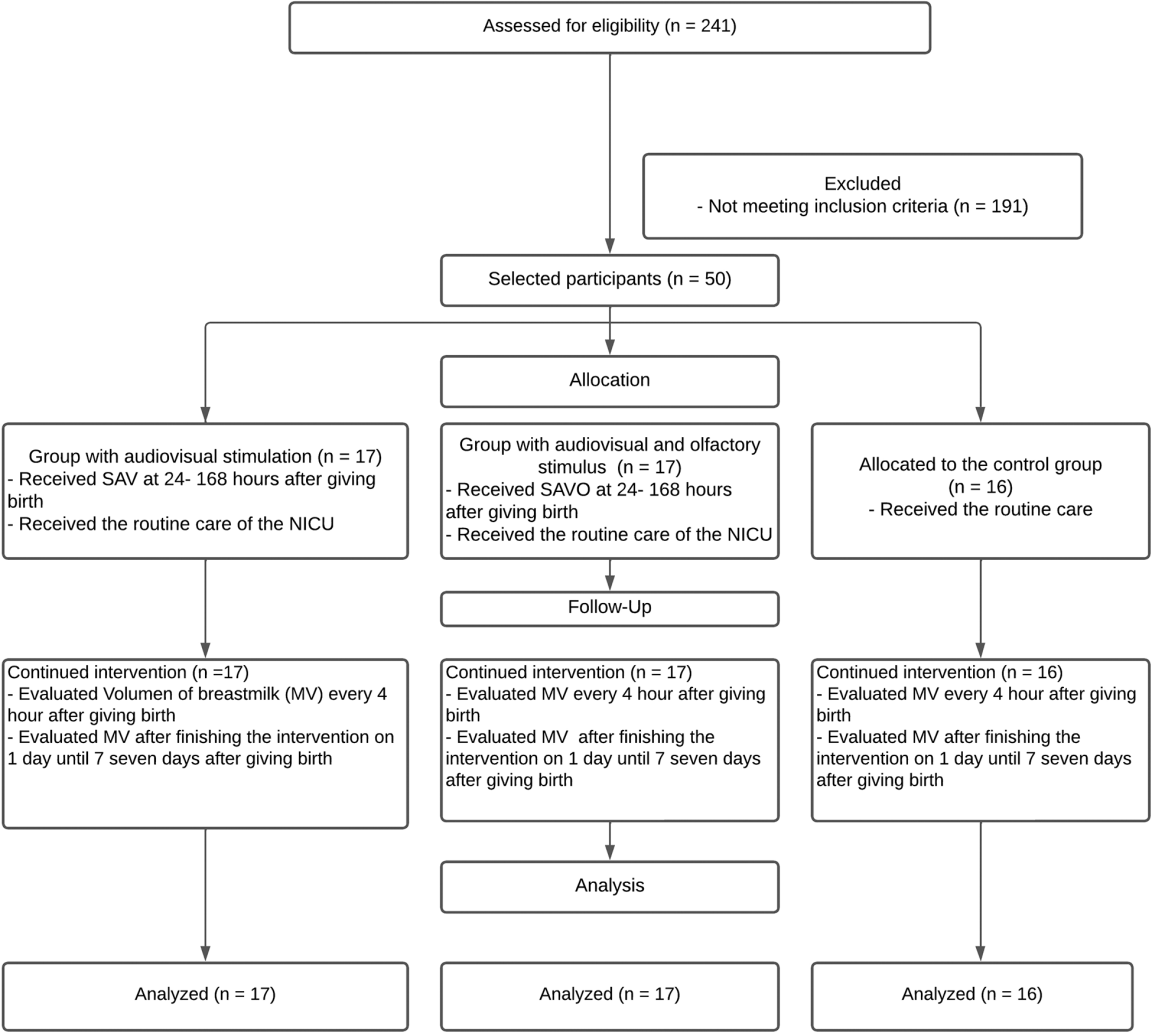
A flow diagram summarizing the participant selection process. SAV, audiovisual stimulation; SAVO, audiovisual and olfactory stimulation; NICU, neonatal intensive care unit; MV, breast milk volume.

In the hospital where this study was conducted, 250 premature births were registered from June to December 2022, among which nine newborns presented malformations. According to the inclusion criteria, the mothers were selected in the delivery room. They were asked to sign the informed consent form to confirm their participation. Of the 241 potential participants, 191 mothers did not meet the inclusion criteria. Therefore, the sample was reduced to 50 participants who were recruited and randomly assigned (online random number generator) (35, 36) to three groups: 17 mothers in the visual-auditory stimulation (SAV) group, 17 in the visual-auditory olfactory stimulation (SAVO) group, and 16 mothers who received general care (control group) (see Figure 1).

### 2.3 Data collection

Two instruments were used to collect data. The first instrument was a questionnaire to collect personal data from the participants (demographics and obstetric history). The second instrument was a form to record daily mother's own milk production. The maternal data collected in the questionnaire included age, marital status, education level, occupation, number of children, comorbidities, mode of delivery, and infant information such as gestational age, sex, birth weight, and Apgar. Sampling was performed for 15 min on each breast for seven consecutive days. All groups in this study received general care from the NICU staff, including educational sessions to teach participants about the importance of mother's own milk, breast massage before extraction, and guidelines on the safe method of expressing and storing breast milk.

The stimuli used in this study took place only in the area designated as a lactation room, an environment designed for the extraction and collection of mother's own milk within the facilities of the Goyeneche Hospital, the stimuli were carried out once a day.

The SAV and SAVO groups watched a video with images and sounds of babies breastfeeding, smiling and crying during the extraction of mother's own milk. The above-mentioned video served as an audiovisual stimulus that was previously validated by two health professionals (a neonatal intensive care specialist and a neonatal nurse) and an expert in social communication. The Delphi method was used and an Aiken V coefficient of 1 was obtained. The video is about 10 min long and includes instrumental music and neonatal crying. The video begins by showing a newborn's hands, feet, and face, then shows babies being breastfed, and ends with an image of a calm newborn. In the video, we included only the faces of healthy babies who were breastfeeding, relaxed, or crying, and excluded the faces of mothers and hospitalized newborns. The video was displayed on a high-definition (HD) 65-inch television screen.

During human milk extraction, the SAVO group, in addition to watching the video, also received an olfactory stimulus consisting of inhaling for approximately 3 min a cotton swab previously rubbed on the newborn's neck, armpits, and groin. NICU staff obtained the cotton swab after the neonate was bathed. They then placed the swabs individually in a hermetically sealed bottle. The control group received only general care from the NICU staff. The amount of milk collected from both breasts was recorded for seven consecutive days after delivery. It is noteworthy that throughout the process of stimulation and milk extraction, the mothers selected for this study did not directly breastfeed their newborns.

### 2.4 Data analysis

In the case of the statistical analysis of the selected sample of mothers (Figure 1), the power analysis was taken as a reference, according to a comparative study between the effectiveness of massage and the use of hot compresses on the breasts to stimulate milk production, which considers a power of 80% and a significance of 5% (37). Descriptive statistics (averages, standard deviations) are used for analysis of sample characteristics, and inferential statistics are used for analysis of continuous variables for estimation of normality. To evaluate the differences between the groups, they were compared using the ANOVA test for continuous data with the use of Tukey's *post hoc* test and the Pearson's *χ*^2^ test for categorical data. Additionally, multinomial logistic regression analysis was conducted. All *p*-values were two-tailed and significance was set at alpha <0.05. The JAMOVI statistical program, version 2.3.21, was used to analyze all data.

## 3 Results

### 3.1 Demographic and obstetric data

Demographic data such as age, marital status, education level, occupation, number of children, and obstetric data such as mode of delivery, Apgar, and birth weight were not significantly different between SAV, SAVO, and the control group (Table 1).

**Table 1:** Demographic and obstetric data of participants.

Demographic and obstetric data	Control group		SAV		SAVO		*F*/*χ*^2^	*p-*value
	(*n* = 16)		(*n* = 17)		(*n* = 17)			
	*n*	%	*n*	%	*n*	%		
Age (years) mean	29.44		27.12		28.29		0.48^a^	0.62
Marital status								
Single	1	6.25	4	23.53	2	11.76		
Married	4	25.00	1	5.88	5	29.41		
Cohabiting	9	56.25	12	70.59	9	52.94	6.87^b^	0.31
Separated/divorced	2	12.50	0	0.00	1	5.88		
Widow	0	0.00	0	0.00	0	0.00		
Level of education								
Uneducated	0	0.00	0	0.00	0	0.00		
Primary school	1	6.25	3	17.65	0	0.00		
High school	8	50.00	9	52.94	8	47.06	6.34^b^	0.39
Technical college	6	37.50	4	23.53	6	35.29		
Graduate university	1	6.25	1	5.88	3	17.65		
Employment status								
Unemployed	0	0.00	0	0.00	0	0.00		
Student	9	56.25	8	47.06	8	47.06		
Self-employed	5	31.25	8	47.06	7	41.18	1.20^b^	0.88
Employed	2	12.50	1	5.88	2	11.76		
Number of children								
Single parent	6	37.50	6	35.29	7	41.18		
Multipara	7	43.75	11	64.71	10	58.82	7.62^b^	0.11
Grand multipara	3	18.75	0	0.00	0	0.00		
Type of delivery								
Eutopic delivery	4	25.00	4	23.53	3	17.65		
Labor dystocia	12	75.00	13	76.47	14	82.35	0.30^c^	0.86
Apgar mean								
1 min apgar score	6.94		8.06		7.53		2.46^a^	0.10
5 min apgar score	8.56		8.88		8.71		1.25^a^	0.30
Birth weight (grams) mean	1,895		2004		2,084		0.295^a^	0.75
Early initiation of breastfeeding (within 1 h of birth)								
No	12	24.0	11	22.0	11	22.0	0.089^b^	0.54
Yes	4	8.0	6	12.0	6	12.0		

Apgar, Appearance, Pulse, Grimace, Activity, and Respiration; SAV, audiovisual stimulation; SAVO, audiovisual and olfactory stimulation.

^a^ One-way ANOVA.

^b^ Likelihood ratio.

^c^ Rho Spearman.

### 3.2 Mother's own milk volume according to stimulus type during days 1–7 postpartum, between the control group, SAV, and SAVO (*N* = 50)

**Figure 2:**
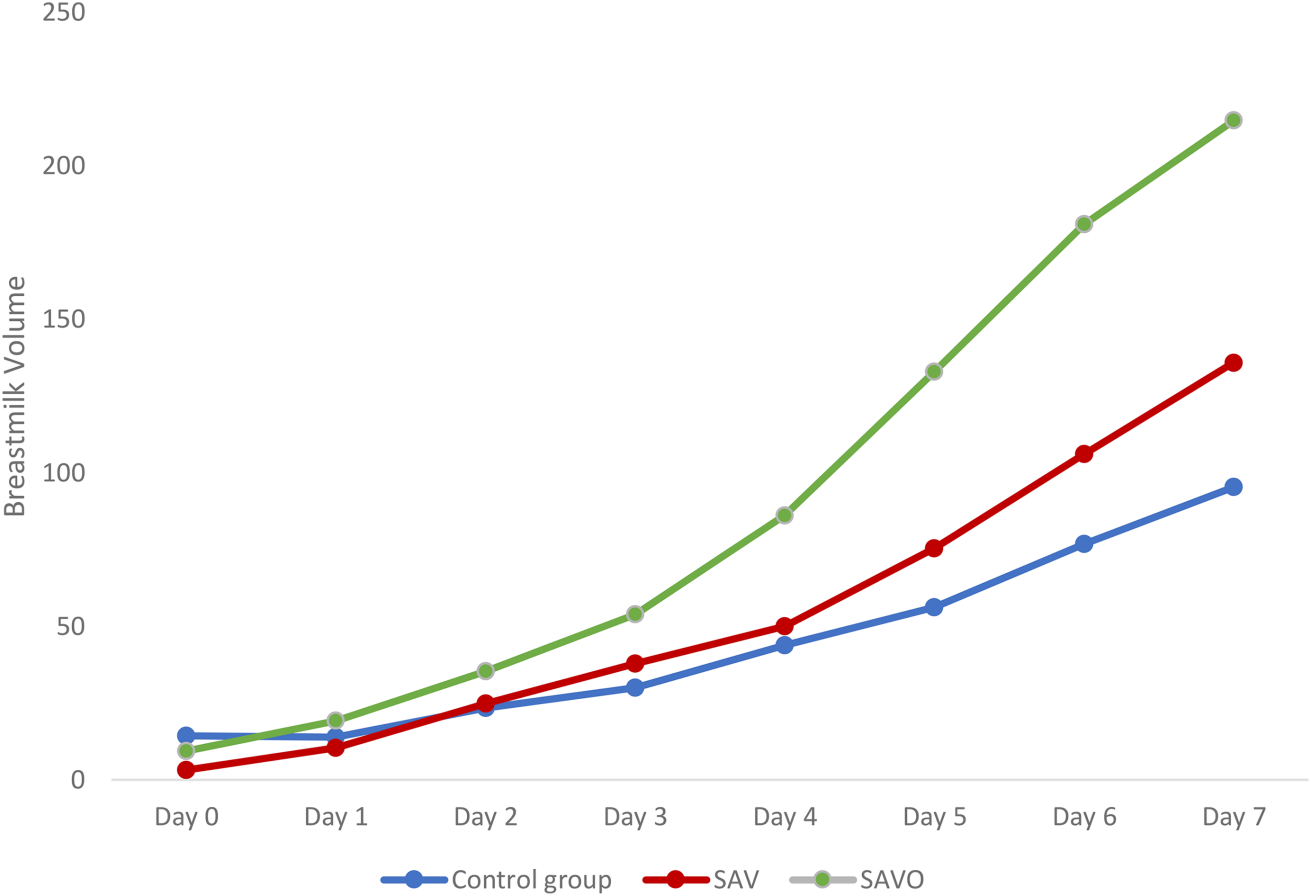
Comparison of breast milk volume between the control group, SAV and SAVO.

Depending on the type of stimulation, an increase in mother's own milk volume was observed, with a greater volume from day 4 to day 7 (Table 2) in the SAV and SAVO stimulation groups compared to the control group (Figure 2).

**Table 2:** Comparison of breast milk volume between control group, SAV and SAVO, days 1–7 postpartum (*N* = 50).

Group	Day 0		Day 1		Day 2		Day 3		Day 4		Day 5		Day 6		Day 7	
	Mean	SD	Mean	SD	Mean	SD	Mean	SD	Mean	SD	Mean	SD	Mean	SD	Mean	SD
Control	14.3	17.7	13.9	17.9	23.4	23.3	30	28.3	43.8	34.7	56.1	33.5	76.9	38.6	95.3	32.5
SAV	3.18	5.51	10.4	12.3	24.8	23.8	37.8	25.3	50	31	75.3	35.3	106	64.9	136	101
SAVO	9.35	10.7	19.2	23.5	35.3	32.9	53.8	35.6	86.2	62.3	133	113	181	152	215	168

### 3.3 One-way ANOVA for between-subject and within-subject comparisons of milk volume between the SAV, SAVO, and control groups

According to mother's own milk volume, significant variance is observed between study groups on day 0 (4 h postpartum) and from day 3 to day 7 (Table 3).

**Table 3:** One-way ANOVA to compare milk volume, between subjects and within subjects between the SAV, the SAVO and the control groups.

Comparison		Sum of squares	Quadratic mean	*F*	Significance^a^
Day 0	Between groups	1,018.027	509.014	3.402	0.042
	Within groups	7,031.353	149.603		
Day 1	Between groups	670.377	335.189	0.981	0.383
	Within groups	16,060.103	341.704		
Day 2	Between groups	1,408.883	704.441	0.959	0.391
	Within groups	34,537.938	734.85		
Day 3	Between groups	4,900.179	2,450.089	2.704	0.077
	Within groups	42,584.941	906.063		
Day 4	Between groups	17,569.029	8,784.515	4.319	0.019
	Within groups	95,601.471	2,034.074		
Day 5	Between groups	53,306.899	26,653.45	5.234	0.009
	Within groups	2,39,350.221	5,092.558		
Day 6	Between groups	95,776.015	47,888.007	4.875	0.012
	Within groups	4,61,681.985	9,823.021		
Day 7	Between groups	1,22,411.768	61,205.884	4.572	0.015
	Within groups	6,29,158.732	13,386.356		

^a^ Significance level at 0.05.

There was a highly significant difference (*p* < 0.05) in the increase of mother's own milk volume in the SAVO group over the 7-day period (Tukey's posthoc test) (Table 4).

**Table 4:** Multiple comparisons regarding the type of stimulation (SAV, SAVO and the control group) in the average volume of breast milk, over a period of 7 days.

Group comparison	Mean (SD)			Mean difference	SE	*F*	*P*
	SAVO group	SAV group	Control group				
	*n* = 17	*n* = 17	*n* = 16				
SAVO with SAV	91.5 (68.3)	55.4 (29.6)		−36.1	0.69	4.93	0.064
SAVO with control	91.5 (68.3)		44.2 (24.1)	−47.3	0.84		0.012
SAV with control		55.4 (29.6)	44.2 (24.1)	−11.2	0.77		0.759

In the multinomial logistic regression analysis, the audiovisual and olfactory stimulation (SAVO) group was more likely to have an increase in average mother's own milk volume than the control group. No significant changes were observed in the audiovisual stimulation and control groups. There were also no significant differences between the SAV and SAVO groups (Table 5).

**Table 5:** Multinomial logistic regression according to stimulus and average milk volume.

Group	Estimator^a^	SE^b^	*Z* ^c^	*p*	OR	IC 95%	
SAV—control group	0.016	0.0142	1.123	0.261	1.016	0.9881	1.045
SAVO—control group	0.0319	0.0144	2.211	0.027	1.032	1.0036	1.062
SAVO—SAV	0.0159	0.00924	1.718	0.086	1.016	0.9978	1.03

IC, confident interval; OR, odds ratio.

Model: AIC = 108, *R*^2^ McF = 0.0901, *p* < 0.05.

^a^ Constant coefficient of the logistic regression.

^b^ Standard error.

^c^ Measures the distance between the estimated coefficient and the null.

On the other hand, the multivariate linear regression analysis between the sociodemographic and obstetric data of the mothers, the type of stimulus and the volume of milk production showed that the relationship between the age of the mother and the multisensory stimulus could intervene in the production of mother's own milk, especially during the first two days (Table 6).

**Table 6:** Multivariate linear regression for sociodemographic and obstetric data according to the volume of breast milk production.

Origin	Dependent variable	Sum of squares type III	DF	Mean square	*F*	Significance
Adjusted model	Day 0	3,893.556^a^	21	185.407	1.249	.287
	Day 1	9,241.576^b^	21	440.075	1.645	.108
	Day 2	19,840.478^c^	21	944.785	1.642	.109
	Day 3	23,939.644^d^	21	1,139.983	1.356	.223
	Day 4	60,968.653^e^	21	2,903.269	1.557	.136
	Day 5	1,60,460.019^f^	21	7,640.953	1.618	.116
	Day 6	2,92,044.657^g^	21	13,906.888	1.467	.170
	Day 7	4,12,037.737^h^	21	19,620.845	1.618	.116
Stimulation vs. mother's age	Day 0	161.124	3	53.708	.362	.781
	Day 1	3,384.022	3	1,128.007	4.217	.014
	Day 2	6,889.859	3	2,296.620	3.993	.017
	Day 3	3,506.734	3	1,168.911	1.390	.266
	Day 4	14,240.719	3	4,746.906	2.546	.076
	Day 5	9,171.626	3	3,057.209	.648	.591
	Day 6	30,517.425	3	10,172.475	1.073	.376
	Day 7	71,640.433	3	23,880.144	1.969	.141
Stimulation vs. marital status	Day 0	290.000	3	96.667	.651	.589
	Day 1	1,170.901	3	390.300	1.459	.247
	Day 2	2,399.310	3	799.770	1.390	.266
	Day 3	2,781.950	3	927.317	1.103	.364
	Day 4	8,867.649	3	2,955.883	1.585	.215
	Day 5	5,349.478	3	1,783.159	.378	.770
	Day 6	11,182.766	3	3,727.589	.393	.759
	Day 7	11,678.976	3	3,892.992	.321	.810
Stimulation vs. mother's level of education	Day 0	249.739	3	83.246	.561	.645
	Day 1	1,528.211	3	509.404	1.905	.152
	Day 2	3,898.211	3	1,299.404	2.259	.103
	Day 3	1,171.622	3	390.541	.464	.709
	Day 4	3,638.633	3	1,212.878	.651	.589
	Day 5	16,075.698	3	5,358.566	1.135	.352
	Day 6	29,563.338	3	9,854.446	1.040	.390
	Day 7	14,926.963	3	4,975.654	.410	.747
Stimulus vs. employment	Day 0	63.022	3	21.007	.142	.934
	Day 1	511.625	3	170.542	.638	.597
	Day 2	147.117	3	49.039	.085	.968
	Day 3	1,585.365	3	528.455	.628	.603
	Day 4	618.075	3	206.025	.111	.953
	Day 5	22,914.347	3	7,638.116	1.618	.208
	Day 6	32,515.745	3	10,838.582	1.143	.349
	Day 7	25,267.459	3	8,422.486	.695	.563
Stimulus vs. number of children	Day 0	263.340	3	87.780	.591	.626
	Day 1	372.804	3	124.268	.465	.709
	Day 2	1,260.493	3	420.164	.730	.543
	Day 3	1,389.152	3	463.051	.551	.652
	Day 4	722.533	3	240.844	.129	.942
	Day 5	5,601.936	3	1,867.312	.396	.757
	Day 6	6,993.697	3	2,331.232	.246	.864
	Day 7	11,048.214	3	3,682.738	.304	.822
Stimulus vs. delivery type	Day 0	144.757	3	48.252	.325	.807
	Day 1	218.665	3	72.888	.273	.845
	Day 2	1,216.143	3	405.381	.705	.557
	Day 3	1,685.480	3	561.827	.668	.579
	Day 4	9,796.012	3	3,265.337	1.751	.179
	Day 5	20,566.263	3	6,855.421	1.452	.249
	Day 6	42,636.508	3	14,212.169	1.499	.236
	Day 7	42,851.376	3	14,283.792	1.178	.336
Stimulation vs. early initiation of breastfeeding	Day 0	991.740	3	330.580	2.227	.107
	Day 1	526.062	3	175.354	.656	.586
	Day 2	287.380	3	95.793	.167	.918
	Day 3	559.507	3	186.502	.222	.880
	Day 4	7,029.460	3	2,343.153	1.257	.308
	Day 5	28,932.153	3	9,644.051	2.043	.131
	Day 6	65,473.426	3	21,824.475	2.302	.099
	Day 7	91,552.002	3	30,517.334	2.517	.079

^a^ *R*^2^ = .484 (*R*^2^ adjusted = .096).

^b^ *R*^2^ = .552 (*R*^2^ adjusted = .217).

^c^ *R*^2^ = .552 (*R*^2^ adjusted = .216).

^d^ *R*^2^ = .504 (*R*^2^ adjusted = .132).

^e^ *R*^2^ = .539 (*R*^2^ adjusted = .193).

^f^ *R*^2^ = .548 (*R*^2^ adjusted = .210).

^g^ *R*^2^ = .524 (*R*^2^ adjusted = .167).

^h^ *R*^2^ = .548 (*R*^2^ adjusted = .209).

## 4 Discussion

The present study evaluated the effectiveness of using multisensory stimuli (audiovisual and olfactory) to increase milk production in mothers of preterm infants. The research was based on the neuroscientific basis of the activation of oxytocin, the main hormone in mother's own milk production, as demonstrated by Uvnäs-Moberg et al. (32), who found that this hormone can be activated by non-noxious sensory stimulation (PVN).

In this context, neuroscience (38) emphasizes that external stimuli from the environment intervene in the activation of the limbic system (39) and take advantage of the integration of different sensory channels (kinesthetic, tactile, visual, gustatory, olfactory, auditory and vestibular) for the development of a comprehensive experience (40). The simultaneous use of the senses creates a stimulating experience that effectively captures the recipient's attention and produces more intense emotions. As a result, information retention is enhanced (41) and a lasting experience is generated, creating an emotional connection with the recipient (31). Furthermore, a study by Rolls et al. (38), reported a link between visual, olfactory and auditory stimuli and the learning process, as both the amygdala and the hippocampus interact with the emotion produced by the stimulus, generating learning that is demonstrated in social and emotional behavior (42).

The literature found so far includes studies with *ad hoc* protocols of multisensory stimulation of preterm and term infants in the areas of mother's own milk production (43), weight gain, length of hospital stay (44) and neuromotor development (45, 46). However, we did not find any studies on mothers of preterm infants related to measurement of milk volume.

It is worth noting that there are only three human milk banks in Peru, none of which are located in the city of this study (Arequipa). However, the Neonatal Intensive Care Unit where the research took place has highly specialized personnel who take care of premature babies and their nutritional needs. Similarly, a multidisciplinary team educates mothers about the importance of feeding their milk to their babies to promote their growth and development.

The results of the present study suggest that the group of mothers who received visual-auditory olfactory stimulation (SAVO) positively increased the volume of milk production (Table 2). This increase could be due to the hormonal role in pregnancy and childbirth. During pregnancy, hormones intervene to facilitate childbirth (47). According to Larsen et al. (48), prolactin regulates neurogenesis in the mother's brain during pregnancy, creating new neuronal networks in the lateral subventricular zone of the brain that enhance the sense of smell. Similarly, Uvnäs-Moberg et al. (32), and Olza et al. (47), reported that oxytocin affects neuroendocrinological, physiological, and psychological processes during labor, delivery, and the early postpartum period and determines maternal behavior.

On the other hand, our study found an increase in milk volume on day 4 in mothers who received visual, auditory, and olfactory stimulation, in contrast to the control group (Table 3). Therefore, it was suggested that multisensory stimulation may positively influence milk production in mothers of preterm infants from the fourth day of lactation. These findings were in agreement with Yu et al. (18), who indicated that lactogenesis II is delayed in mothers of preterm infants, and milk volume is lower in the first 24 h after birth. Therefore, for a higher increase in volume, the stimuli should be applied 48 h after delivery.

Conversely, Mullen et al. (19), suggest that early breastfeeding (before 6 h after birth) is a primary factor in milk production in mothers of preterm infants. In this study, there was no significant difference in the type of multisensory stimulus used (Table 6). Yu et al. (18), found that late lactogenesis II was associated with the type of delivery, particularly cesarean delivery. However, Heller et al. (49), pointed out that this information is inconclusive for preterm birth. Regarding the type of delivery, there was no significant differences between stimulation type and milk production (Table 6).

The parity of the mother is a controversial factor, as some studies, such as Zachariassen et al. (50), suggest a significant association between being primiparous and successful initiation of breastfeeding. On the contrary, Maas-trup et al. (51), suggests that multiparity is associated with successful breastfeeding. However, in this study, no significant differences were found in stimulation and mother's own milk volume with parity of the mother. However, a significant difference was found between multisensory stimulation, mother's own milk volume on days 1 and 2, and maternal age. There were no conclusive studies on the association between maternal age and mother's own milk production. However, we found evidence of an association between maternal age and successful breastfeeding after discharge of a preterm infant. In this regard, Pineda et al. (52), found that young mothers were less successful in maintaining breastfeeding. Similarly, Casey et al. (53), indicated that maternal age is a predictor of failure to maintain exclusive breastfeeding.

Other sociodemographic factors, such as maternal marital status, education level, and occupation, did not show significant differences in stimulation with mother's own milk volume. Nevertheless, several studies have demonstrated the association of the above-mentioned variables with the success of maintaining exclusive breastfeeding in preterm infants after discharge from hospital (49, 52).

According to Mitha et al. (54), the factors that promote breastfeeding in preterm infants in the NICU are skin-to-skin contact using the kangaroo position during the first week of life, continuous information to mothers about the importance of breastfeeding, expressing their milk within 6 h of birth, the provision of a designated room (in the NICU) for expressing their milk, and support from staff trained in breastfeeding. In contrast, Heller et al. (49), did not find a significant association between the duration of breastfeeding in preterm infants and health care provider information, availability of a lactation room and staff trained in breastfeeding. Therefore, further studies are needed to determine the relationship between these factors and mother's own milk production.

Due to the hospital's COVID-19 protocol, postpartum mothers with preterm infants in the NICU were not able to perform skin-to-skin contact (kangaroo method). The absence or delay of this method is considered to be a risk factor for the successful establishment of exclusive breastfeeding at discharge (54–56). Therefore, it would be essential to implement the multisensory stimuli proposed in this study with mothers who use the kangaroo method, in order to evaluate its impact on mother's own milk production.

Despite the limitations imposed by the context of the COVID-19 pandemic, the study was rigorously conducted. Thus, the limitations did not affect our findings.

Finally, the results found in this study may have relevant clinical implications for neonatal intensive care units that are faced with insufficient mother's own milk to feed premature infants and lack access to human milk banks. The incorporation of multisensory stimulation would represent an innovative approach to mother's own milk production and mother-infant care.

## 5 Conclusion

Multisensory stimulation in postpartum mothers of preterm infants may influence an increase in mother's own milk production, according to the findings of this study. However, more research is needed in this area to clarify the reported findings.

## Data Availability

The datasets presented in this study can be found in online repositories. The names of the repository/repositories and accession number(s) can be found in the article/Supplementary Material.
